# Efficacy and safety of PD-1/PD-L1 inhibitors in the treatment of recurrent and refractory ovarian cancer: A systematic review and a meta-analysis

**DOI:** 10.3389/fphar.2023.1111061

**Published:** 2023-03-13

**Authors:** Siyuan Zeng, Daju Liu, Yongai Yu, Lei Zou, Xianyu Jin, Bing Liu, Lifeng Liu

**Affiliations:** ^1^ Department of Obstetrics and Gynecology, Dalian Municipal Central Hospital, Dalian, China; ^2^ Dalian municipal Central Hospital, China Medical University, Shenyang, China

**Keywords:** recurrent/refractory ovarian cancer, PD-1/PD-L1 inhibitors, immunotherapy, immunocheckpoint inhibitors, meta-analysis

## Abstract

**Objective:** To explore the efficacy and safety of PD-1/PD-L1 inhibitors in treating recurrent/refractory ovarian cancer (OC).

**Methods:** The online databases, including PubMed, Embase and Cochrane Library, were searched for relevant literatures on exploring the efficacy and safety of PD-1/PD-L1 inhibitors in the treatment of recurrent/refractory OC. The keywords are as follows: Ovarian neoplasms, programmed death receptor, PD-1, PD-L1, immunotherapy, and immune checkpoint inhibitor. Furthermore, qualified studies were screened for further meta-analysis.

**Results:** In this study, 11 studies (990 patients) were analyzed to evaluate the efficacy of PD-1/PD-L1 inhibitors in the treatment of recurrent/refractory OC. The combined results proved that the objective response rate (ORR) was 6.7%, 95% CI (4.6%,9.2%), disease control rate (DCR) was 37.9%, 95% CI (33.0%, 42.8%), median overall survival (OS) was 10.70 months, 95% CI (9.23, 12.17), and median progression free survival (PFS) was 2.24 months, 95% CI (2.05, 2.43). In addition, in terms of the safety of patients suffering from recurrent/refractory OC and receiving PD-1/PD-L1 inhibitors, the combined treatment related adverse events (TRAEs) were 70.9% (61.7%–80.2%), and the combined immune related adverse events (iAEs) were 29%, 95% CI (14.7%, 43.3%).

**Conclusion:** In patients with recurrent/refractory OC, PD-1/PD-L1 inhibitors were used alone and there was no obvious evidence of improved efficacy and survival. As for safety, the incidences of TRAEs and iAEs are high, so PD1/PD-L1 inhibitors should be applied according to individual conditions.

**Clinical Trial Registration:** https://www.crd.york.ac.uk/PROSPERO/display_record.php?RecordID=367525, identifier CRD42022367525.

## Introduction

Ovarian cancer (OC) is the second leading cause of female gynecological cancer death worldwide (Bray et al., 2020). It was estimated that 313,959 people were diagnosed with OC and 207,252 people died of OC in the world in 2020 (Global, 2020; Siegel et al., 2021). The early symptoms of OC were insidious, and most of the patients were advanced at the time of treatment. Traditional treatment for OC was mainly surgery plus adjuvant chemotherapy, while the emergence of chemotherapy resistance affects the prognosis of patients to a certain extent, so the median survival period of advanced/recurrent epithelial OC is only 14.6 months (Shimokawa et al., 2018). The latest concept pointed out that the treatment for OC can be evolved into the treatment for chronic diseases, and the future treatment mode of OC will gradually move towards the trend of combined treatment, including surgery, chemotherapy, endocrine therapy, immunotherapy, and other methods (Ovarian, 2022).

Tumor immunotherapy is the fourth anti-tumor therapy after surgery, chemotherapy, and radiotherapy. In the tumor microenvironment, tumor cells express corresponding ligands, thus leading to T cell dysfunction, which enables tumor cells to escape the surveillance and clearance of the immune system. Targeted drugs against cytotoxic T-lymphocyte associated protein 4 (CTLA4) and programmed cell death receptor 1 (PDCD1, also known as PD-1)/programmed cell death receptor ligand 1 (PDCD1LG1, also known as PD-L1) play an anti-tumor effect by relieving tumor cells’ inhibition of T cell function (O'Donnell et al., 2017). In recent years, immune checkpoint inhibitors (ICI), as the most common immunotherapy, have brought hope for treating malignant tumors (Boustani et al., 2021).

On 17 August 2021, Food and Drug Administration (FDA) approved Dostarlimab-gxly (JEMPERLI) for adult patients with dMMR, relapsed, or advanced solid tumors who have progressed on or after prior therapy and had no satisfactory alternative therapy. (Markham, 2021). In September 2021, the National Comprehensive Cancer Network (NCCN) guidelines were recommended for use in the guidelines for uterine tumors and ovarian cancer. In 2022, the NCCN guidelines recommended that the indications for using PD-1/PD-L1 inhibitors in advanced/recurrent OC mainly include: Tumor tissue is in deficient mismatch repair (dMMR) or microsatellite instability high (MSI-H) state, and tumor mutation burden-high (TMB-H ≥10 muts/MB) (Ovarian, 2022).

The effects of PD-1/PD-L1 inhibitors, such as Nivolumab, Pembrolizumab, Avelumab, and Atezolizumab, have been confirmed by clinical studies (Brahmer et al., 2012; Hamanishi et al., 2015; Disis et al., 2019; Liu et al., 2019; Matulonis et al., 2019; Normann et al., 2019; Varga et al., 2019; Desai et al., 2020; Hamanishi et al., 2021; Pujade-Lauraine et al., 2021). Most of these tests are designed as single arm tests, and the form is irreparable. Therefore, we conducted a meta-analysis to investigate the efficacy of anti-PD-1/PD-L1 alone in OC.

## Materials and methods

### Searching strategy and selection criteria

This meta-analysis was conducted through the Preferred Reporting Items for Systematic Reviews and Meta-analysis (PRISMA) standard (Moher et al., 2009). The following keywords were searched in PubMed, Embase, Cochrane Library and other online databases: Ovarian neoplasms, programmed death receptor, PD-1, PD-L1, immunotherapy, and immune checkpoint inhibitor. The selection criteria are as follows: 1) The data of observation indicators were complete; 2) The studies were prospective clinical studies, including randomized controlled trials and single arm studies. For clinical controlled trials, only the study group receiving single drug treatment of PD-1/PD-L1 inhibitors was included; 3) Patients with recurrent/refractory OC received single drug treatment of PD-1/PD-L1 inhibitors (Nivolumab, Pembrolizumab, Avelumab, and Atezolizumab); 4) Literature published in English. Exclusion criteria are as follows: 1) Article type: Letters, editorials, expert opinions, case reports and comments; 2) Research without available data; 3) Repetitive publications.

### Data extraction and quality assessment

Two researchers were independently responsible for data extraction, and any differences were resolved by third-party contributors. The following data were extracted using the previously developed data extraction table: 1) Literature related information: Author’s name, research year, research type, total number of people in the study and the corresponding number of people in each group; 2) Study event indicators: Objective response rate (ORR) and disease control rate (DCR), median overall survival (OS) and median progression free survival (PFS), and treatment related adverse events (TRAEs)/immune related adverse events (iAEs).

### Statistical analysis

The combined ORR/DCR/medium OS/median PFS/TRAEs/iAEs were statistically analyzed using Stata version 15.0. Cochran Q test and I^2^ statistical evaluation were used for data heterogeneity assessment. For Q test, *p*-value less than 0.05 indicates significant heterogeneity; For I^2^ statistics, I^2^ values greater than 50% are considered significant heterogeneity. Subgroup analysis was conducted to explore potential sources of heterogeneity. By removing each study and calculating, the sensitivity analysis was used to determine the related effects of individual studies on the combined results. Begg’s and Egger’s test were depicted to assess publication bias. *p* < 0.05 was considered to be statistically significant.

## Results

### Eligible literatures

**FIGURE 1:**
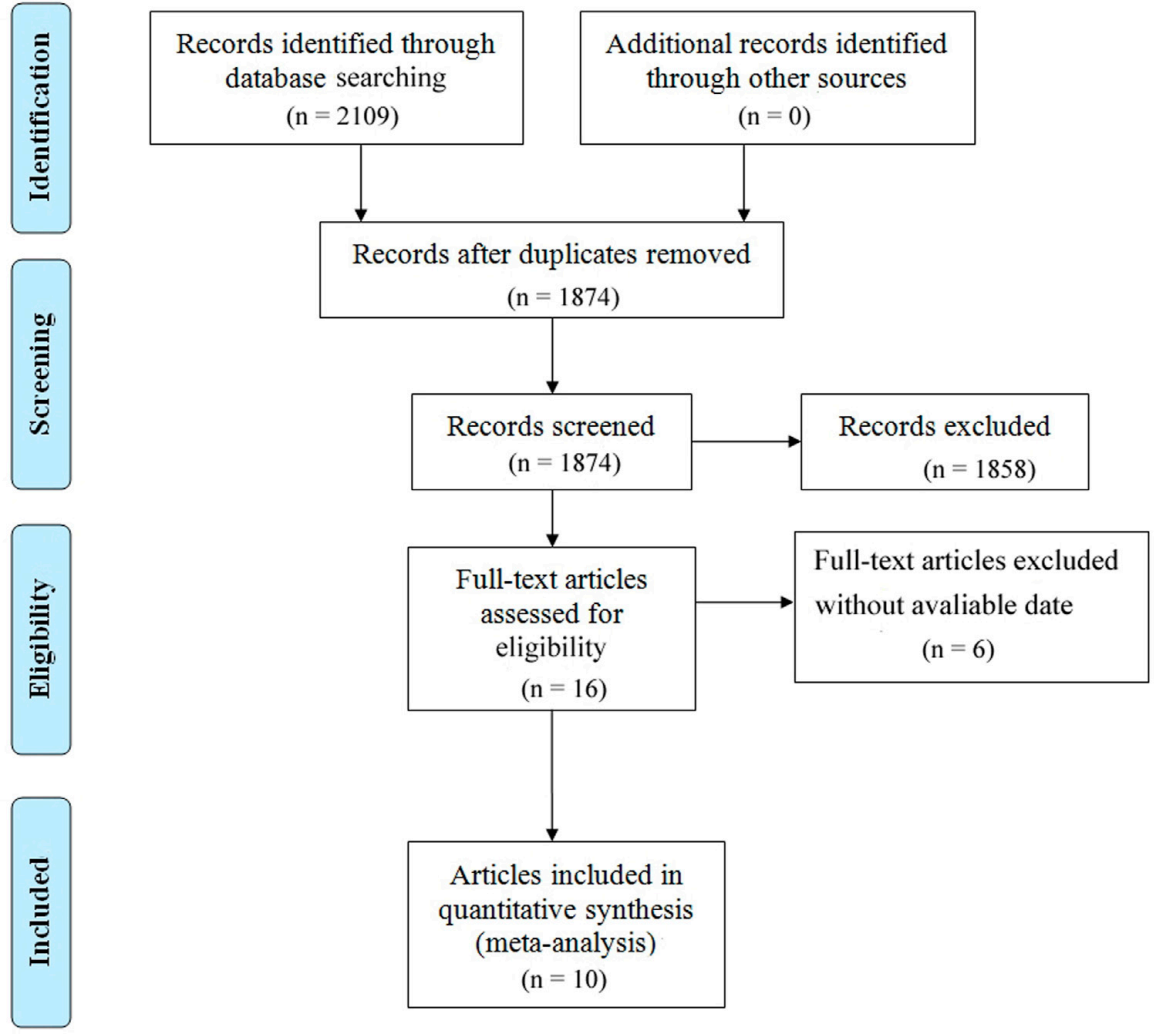
Flow chart of the meta-analysis.

A total of 2,109 articles were initially searched, and 1,874 articles remained after eliminating duplication. By reviewing the title and abstract according to the inclusion and exclusion criteria, 1,858 articles were excluded. Finally, through full text review, 10 articles with 11 researches, involving 990 OC patients, were recruited. PD-1/PD-L1 inhibitors used included Nivolumab (*n* = 4), Pembrolizumab (*n* = 3), Avelumab (*n* = 2), Atezolizumab (*n* = 1), and Tislelizumab (*n* = l). All the PD-1/PD-L1 inhibitors have been approved by FDA. The basic information of eligible research in Table 1. All participants in the 11 studies were diagnosed with recurrent or refractory OC. See the following detailed flow chart (Figure 1). According to the indicators of the Methodological Index for Non-randomized Studies (MINORS) scale, the total score of the quality evaluation is 16 points; 0 point means that the literature was not reported; 1 point refers to that the literature has been reported but the data information was insufficient; 2 points represents that the literature has been reported and provided sufficient information. The results are shown in (Supplementary Table S1).

**TABLE 1:** Characteristics of studies included in this meta-analysis.

Study, year	Sample size	Age	Median follow-up (months)	Drugs	Interventions	Median OS with 95% CI (months)	Median PFS with 95% CI (months)
Brahmer 2012	17	NA	NA	BMS-936559 (Nivolumab)	3 or 10 mg/kg every 2 weeks	NA	NA
Hamanishi 2015	20	Median: 60.0	8	Nivolumab	Every 2 weeks at a dose of 1 or 3 mg/kg	20.0 (7.0-NR)	3.5 (1.7–3.9)
Varga 2018	26	Median: 57.5	15.4	Pembrolizumab	10 mg/kg every 2 weeks for ≤24 months	13.8 (6.7–18.8)	1.9 (1.8–3.5)
Disis 2019	125	Median: 62.0	26.6	Avelumab	Avelumab 10 mg/kg was administered by 1 h intravenous infusion every 2 weeks	11.2 (8.7–15.4)	2.6 (1.4–2.8)
Liu 2019	12	Median: 60.5	7.6	Atezolizumab	Atezolizumab was administered intravenously at 15 mg/kg or 1,200 mg every 3 weeks	11.3 (5.5–27.7)	2.9 (1.3–5.5)
Matulonis 2019 1	285	NA	16.7	Pembrolizumab	Pembrolizumab 200 mg was administered intravenously every 3 weeks	NR (16.8-NR)	2.1 (2.1–2.2)
Matulonis 2019 2	91	NA	17.3	Pembrolizumab	Pembrolizumab 200 mg was administered intravenously every 3 weeks	17.6 (13.3-NR)	2.1 (2.1–2.6)
Normann 2019	18	Median: 61.0	7.5	Nivolumab	Nivolumab 3 mg/kg bodyweight every second week	7.5 (3.75–11.25)	3.75 (3.25–4.25)
Desai 2020	51	Median: 61.0	13.6	Tislelizumab	2 mg/kg administered every 2 weeks	NA	NA
Eric 2021	188	Median: 61.0	18.2	Avelumab	Avelumab 10 mg/kg was administered by 1 h intravenous infusion every 2 weeks	11.8 (8.9–14.1)	1.9 (1.8–1.9)
Hamanishi 2021	157	Median: 58.0	NA	Nivolumab	Nivolumab 240 mg was administered intravenously every 2 weeks	10.1 (8.3–14.4)	2.0 (1.9–2.2)

NA: not acpuired; NR: not reached.

### Efficacy evaluation

**FIGURE 2:**
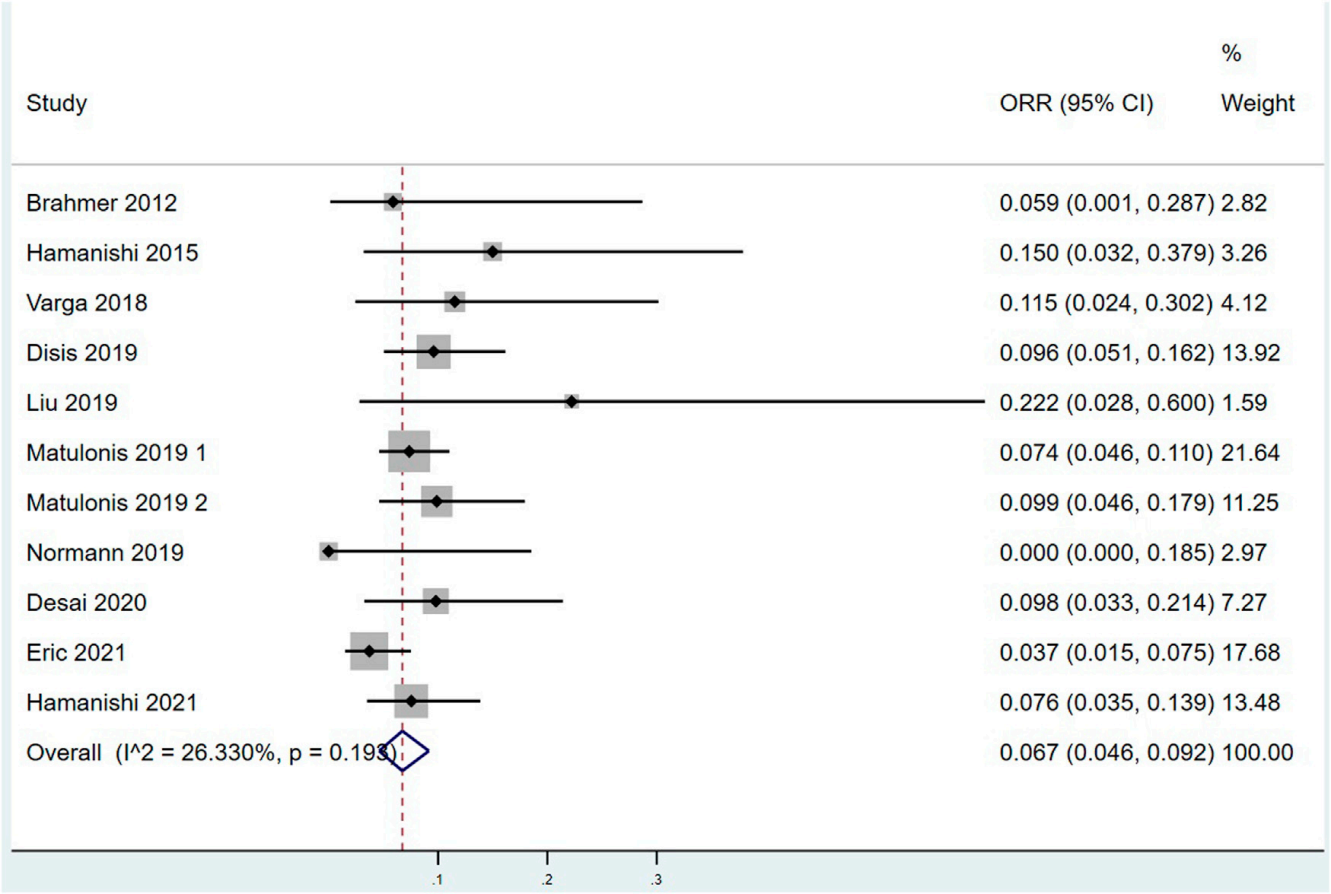
Forest plots of the pooled objective response rate (ORR).

**FIGURE 3:**
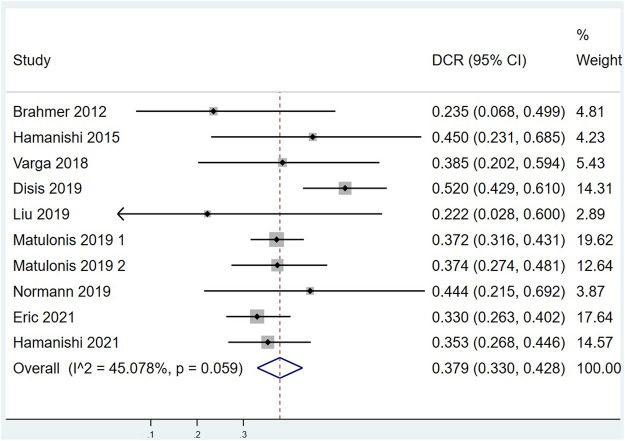
Forest plots of the pooled disease control rate (DCR).

**FIGURE 4:**
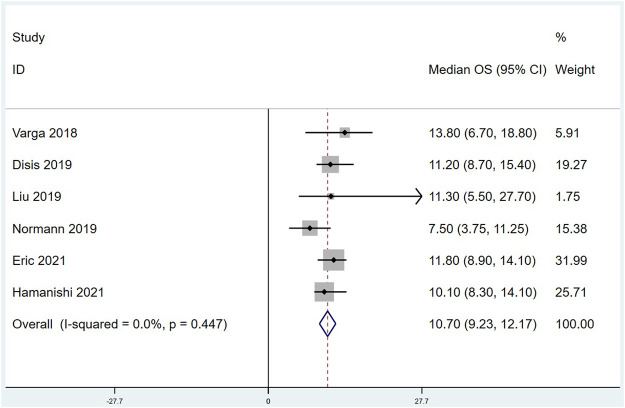
Forest plots of the Summarized median overall survival (OS).

**FIGURE 5:**
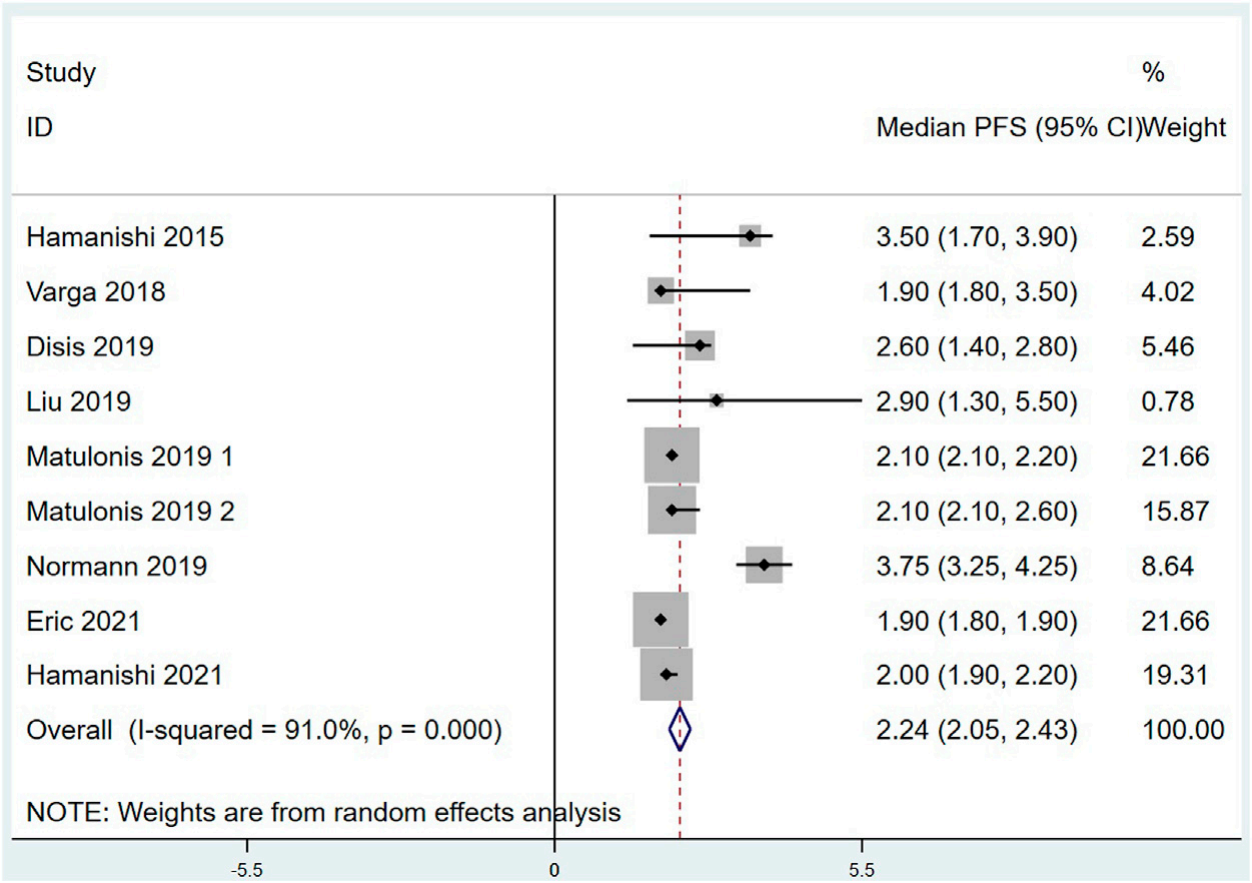
Forest plots of the Summarized median progression free survival (PFS).

Eleven of the included studies made statistics on ORR. The total sample size was 949 cases, with 72 cases of objective response. Meta analysis results are as follows: I^2^ = 26.33%, *p* = 0.193 indicates that there is no heterogeneity among the 11 studies. The fixed effect model was selected, and the combined effect value ORR = 6.7%, 95% CI (4.6%,9.2%), (Figure 2). Ten of the included studies made statistics on DCR. The total sample size was 898 cases, with 342 cases of disease control. Meta analysis results are as follows: I^2^ = 45.078%, *p* = 0.059 refers to that there was moderate heterogeneity among the studies, the random effect model was used with the combined effect value of DCR = 37.9%, 95% CI (33.0%, 42.8%), (Figure 3). Six of the included studies made statistics on the median OS. Meta analysis results are that I^2^ = 0.0%, *p* = 0.447 suggests that there was no significant heterogeneity among the 6 studies, so the fixed effect model was used, with the combined effect value median OS = 10.70 months, 95% CI (9.23, 12.17), (Figure 4). Nine of the included studies made statistics on the median PFS. Meta analysis results are that I^2^ = 91.0%, *p* < 0.001 represents that there was significant heterogeneity among the studies, so a random effect model was used, with the combined effect value median PFS = 2.24 months, 95% CI (2.05, 2.43), (Figure 5).

### Safety assessment

**FIGURE 6:**
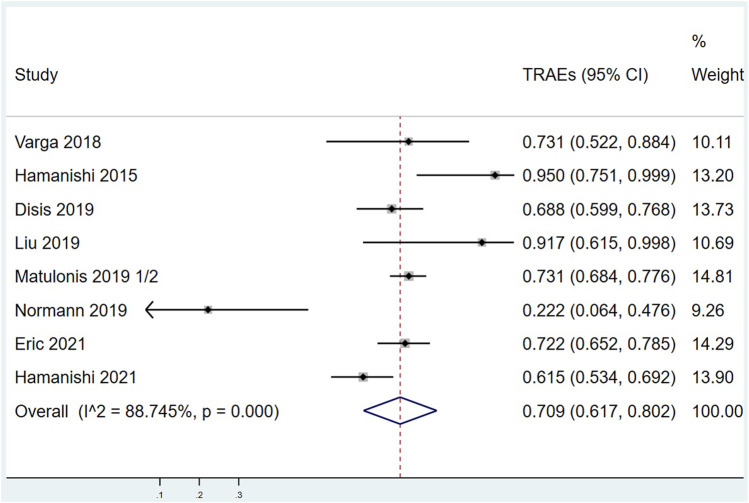
Forest plots of the overall treatment related adverse events rate (TRAEs).

**FIGURE 7:**
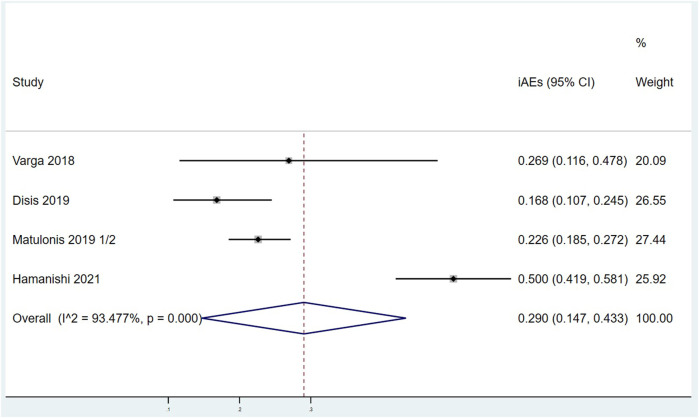
Forest plots of the overall immune related adverse events rate (iAEs).

There were 8 articles that counted the incidence of TRAEs. Meta analysis results are as follows: I^2^ = 88.745%, *p* < 0.001 indicates that there is a certain heterogeneity among the studies, so a random effect model was used, with combined effect values of TRAEs = 70.9%, 95% CI (61.7%, 80.2%), (Figure 6). TRAEs can be divided into mild-moderate TRAEs (Grade 1 = mild, Grade 2 = moderate) and severe TRAEs (Grade 3 = severe, Grade 4 = life-threatening, and Grade 5 = death) according to severity. As shown in Supplementary Figure S1, the pooled mild-moderate TRAEs is 53.0%, 95% CI (44.0%, 62.0%) with huge heterogeneity (I^2^ = 83.817%, *p* < 0.001). The combined effect value of severe TRAEs was 13.3%, 95% CI (8.0%, 18.6%) with significant heterogeneity (I^2^ = 77.433%, *p* < 0.001). There were 4 articles that counted the incidence of iAEs. Meta analysis results are that I^2^ = 93.477%, *p* < 0.001 marks that there is some heterogeneity between the four studies, so a random effect model was used, with combined effect values of iAEs = 29.0%, 95% CI (14.7%, 43.3%), (Figure 7).

### Subgroup analysis

In Table 2, according to Avelumab, Nivolumab, Pembrolizumab, and others, the ORR was divided into four subgroups. Meta analysis results are as follows: the ORR of Avelumab combination effect value is 5.8% (3.4%–8.7%), DCR is 40.0% (34.7%–45.4%), median OS is 11.57 months (9.52–13.63), median PFS is 2.16 months (1.5–2.82), TRAEs is 70.9% (65.9%–75.9%), and iAEs is 16.8% (10.7%–24.5%); Nivolumab combination effect value ORR is 6.1% (1.9%–11.8%), DCR is 35.7% (28.7%–42.8%), median OS is 9.13 months (6.83–11.42), median PFS is 3.05 months (1.66–4.43), TRAEs is 60.6% (27.7%–93.5%), and iAEs is 50.0% (41.9%–58.1%); Pembrolizumab combination ORR is 7.7% (5.2%–10.7%), DCR is 37.3% (32.6%–42.0%), median OS is 13.8 months (7.75–19.85), median PFS is 2.10 months (2.05–2.15), TRAEs is 73.1% (68.4%–77.6%), and iAEs is 22.9% (18.8%–27.0%); Others combination effect value ORR is 10.2% (3.0%–20.1%), DCR is 22.2% (2.8%–60.0%), median OS is 11.30 months (0.20–22.40), median PFS is 2.90 months (0.80–5.00), and TRAEs is 83.2% (71.6%–94.7%).

**TABLE 2:** Summary of effectiveness and safety of different treatment combinations.

	Effect value (95%CI)					
Subgroup	ORR	DCR	OS	PFS	TRAEs	iAEs
Avelumab	5.8% (3.4%–8.7%)	40.0% (34.7%–45.4%)	11.57 months (9.52–13.63)	2.16 months (1.5–2.82)	70.9% (65.9%–75.9%)	16.8% (10.7%–24.5%)
Nivolumab	6.1% (1.9%–11.8%)	35.7% (28.7%–42.8%)	9.13 months (6.83–11.42)	3.05 months (1.66–4.43)	60.6% (27.7%–93.5%)	50.0% (41.9%–58.1%)
Pembrolizumab	7.7% (5.2%–10.7%)	37.3% (32.6%–42.0%)	13.8 months (7.75–19.85)	2.10 months (2.05–2.15)	73.1% (68.4%–77.6%)	22.9% (18.8%–27.0%)
Other	10.2% (3.0%–20.1%)	22.2% (2.8%–60.0%)	11.30 months (0.20–22.40)	2.90 months (0.80–5.00)	83.2% (71.6%–94.7%)	—

### Publication bias and sensitivity analyses

We performed Begg’s and Egger’s tests to assess the presence of publication bias in this study. As displayed in Supplementary Figure S2, publication bias was not significant in studies on ORR (*p* = 0.107), DCR (*p* = 0.592), OS (*p* = 0.707), PFS (*p* = 0.466), TRAEs (*p* = 0.711), and iRAEs (*p* = 0.734) based on Begg’s tests. Similar results were observed based on Egger’s tests (ORR: *p* = 0.06; DCR: *p* = 0.919; OS: *p* = 0.724; PFS: *p* = 0.225; TRAEs: *p* = 0.775; iRAEs: *p* = 0.653) (Supplementary Figure S3). Sensitivity analysis proved that our results were robust (Supplementary Figure S4).

## Discussion

In this study, 11 studies, involving 990 patients, were analyzed to evaluate the efficacy of PD-1/PD-L1 inhibitors in the treatment of recurrent/refractory OC. Our results indicated that in patients with recurrent/refractory OC, PD-1/PD-L1 inhibitors were used alone and there was no obvious evidence for the improvement of efficacy and survival. As for safety, the incidences of TRAEs and iAEs are high, so PD-1/PD-L1 inhibitors should be applied based on individual conditions.

Currently, the 2022 NCCN guidelines show that PD-1/PD-L1 inhibitors are effective in some cases of recurrent epithelial OC (including rare pathological types) and recurrent malignant germ cell tumors, but PD1/PD-L1 inhibitors are not recommended for recurrent malignant sex cord stromal tumors. Therefore, in the case of unsatisfactory effects of surgery, chemotherapy and other treatment methods, immunotherapy can be considered for such patients (Ovarian, 2022). However, the guidelines only recommend that patients with MSI-H/dMMR may benefit from immunotherapy. Although PD-1/PD-L1 inhibitors have made breakthrough progress in the treatment of gynecological tumors, the current immunotherapy effect in OC is still not satisfactory or perfect. The reasons are closely related to the immune escape mechanism of OC and the changes of tumor immune microenvironment (TIME): 1) Recognizing related antigens is weakly developed: Among various cancers, including OC, NY-ESO-1 is considered as a promising and effective target for immunotherapy (Gordeeva, 2018). Some studies revealed that no NY-ESO-1 peptide antigen was found in major histocompatibility complex (MHC-1) or MHC-2 molecules in 42 epithelial OC samples (Schuster et al., 2017). In clinical trials of OC, many solid tumor targets rely on tumor related antigens that have been discovered, such as HER2, WT1, NY-ESO-1, and p53, while they do not all exist on MHC molecules. Meanwhile, these studies has not been proved that the tumor antigens in question do not get processed and presented on MHC-1 or MHC-2 molecules. Therefore, the induction of immune response against these antigens may mislead immune cells, and thus prevent them from attacking tumor cells. 2) Inhibition of antigen presenting cells: Antigen presenting cells include macrophages, dendritic cells, and B lymphocytes. Severe dysfunction of dendritic cells occurs in advanced OC. Cancer cells infiltrate dendritic cells in large quantities and secrete PGE2 and TGF- *β*. By inducing PD-L1 and arginase activity, normal dendritic cells with immune function can be transformed into immunosuppressive cells (Chae et al., 2017). 3) Inhibition of tumor killing immune cells and activation of immunosuppressive cells: Regulatory T cells (Tregs) can inhibit the anti-tumor response of T cells. Tregs negatively regulates anti-tumor response in a direct and indirect manner (Mishra et al., 2021; Puleo and Polyak, 2022). Curiel et al. confirmed that CD4^+^CD25+FOXP3+Tregs specifically inhibited anti-tumor T cells *in vivo* and promoted tumor growth (Curiel et al., 2004). Their existence is correlated with the poor prognosis of OC. Myeloid derived suppressor cells (MDSCs) have a significant ability to inhibit T cell response (Lim et al., 2020), and they increase in OC patients and play an important role in disease progression. 4) The mechanisms of the OC immunosuppressive network include the inhibition of CD8+effector T cells by Tregs, as described previously. Secondly, indoleamine-2, 3-dioxygenase (IDO), an immune regulatory enzyme, induces immune tolerance by locally consuming tryptophan and producing toxic tryptophan metabolites (such as kynurenine), thus resulting in the growth of effector T cells or NK cells in TME that is hindered and inhibits their killing function. IDO inhibitors can improve the anti-tumor efficacy of current chemotherapy or immunotherapy (Zhai et al., 2020); The most classical inhibition pathway is the combination of inhibitory immune checkpoint CTLA4 and PD-1 with ligand PD-L1; in addition, MDSCs and inhibitors, such as TGF- β, also participate in regulating the immunosuppressive network of OC (Odunsi, 2017).

Future research directions of immunotherapy are displayed as follows. Most preclinical and clinical studies focus on recurrent/metastatic/persistent/late unresectable gynecologic tumors (Brahmer et al., 2012; Hamanishi et al., 2015; Disis et al., 2019; Liu et al., 2019; Matulonis et al., 2019; Normann et al., 2019; Varga et al., 2019; Desai et al., 2020; Hamanishi et al., 2021; Pujade-Lauraine et al., 2021). However, more and more evidence supports that it should be used as early as possible when the patients are in generally good condition and the tumor load is small. In recent years, studies have suggested that early application of PD-1/PD-L1 inhibitor in the treatment of triple negative breast cancer and non-small cell lung cancer could benefit patients (Topalian et al., 2020). Another highly concerned treatment direction is combination therapy. Research in OC illustrated that the ORR of the combination of PD-1/PD-L1 inhibitors and chemotherapy was the highest [36% (95% CI: 24%, 51%)], the ORR of the combination of PD-1/PD-L1 inhibitors and anti-angiogenesis therapy was 30% (95% CI: 19%, 44%), and the ORR of the combination of PD-1/PD-L1 inhibitors and poly adenosine diphosphate ribose polymerase (PARP) inhibitors was 17% (95% CI:11%, 26%) (Zhu et al., 2021). However, the combined use of multiple drugs is like a double-edged sword, which brings about a new problem: the toxic and side effects of drugs. Compared with the existing PD-1/PD-L1 inhibitor combined with chemotherapy, this study showed that the single drug treatment of PD1/PD-L1 inhibitor could not significantly reduce the incidence of serious adverse reactions. Therefore, how to achieve the best treatment effect for patients with the least toxic and side effects is an urgent problem to be solved in the combined application of PD-1/PD-L1 inhibitors. It is also one of the research directions in the future to explore more effective predictors. At present, clinical efficacy predictors include dMMR/MSI-H, PD-L1, and TMB-H, but these predictors are not ideal. On the one hand, it is because the ORR of PD-1 inhibitor is poor, even in PD-L1 positive OC patients (Varga et al., 2019). On the other hand, KEYNOTE-100 shows a low MSI rate in OC (Matulonis et al., 2019), and the TMB of OC patients is also very low.

Moreover, before using immunotherapy, we should carefully consider the patient’s sociological factors, lifestyle, metabolic disorders, and other variables, aiming to obtain the best treatment results. In this study, it showed that race, obesity, smoking, exercise, and drinking habits affect the effectiveness of immunotherapy, while diabetes and hypertension are the results of immunotherapy, rather than the causes. Hormone signaling also affects prostate cancer, endometrial cancer, OC, and colon cancer. It is imperative to determine the hormone response profile of individual tumor in the context of ICI therapy (Deshpande et al., 2020).

Zhu J et al. pointed out that PD-1/PD-L1 inhibitors alone have limited efficacy in OC. PD-1/PD-L1 inhibitor combined with chemotherapy can be recommended for further research. Compared with their research, this study further explored the safety of immunotherapy, the immune escape mechanism of OC and the efficacy of various types of PD-1/PD-L1 inhibitors on OC (Zhu et al., 2021).

This study has some limitations. First, most of the articles included were non-comparable, and include phase I–III clinical studies, which makes this study have certain heterogeneity. Second, PD-1/PD-L1 inhibitors are different in the study, which inevitably leads to deviation. Third, there is not enough data to evaluate the patient’s Body Mass Index, allergy history, race, drinking history, smoking history, and other characteristics.

## Conclusion

To our knowledge, this meta-analysis is the first to focus on the efficacy of PD-1/PD-L1 inhibitors alone in recurrent/refractory OC, which is timely and necessary. According to this study, the efficacy and safety of PD-1/PD-L1 inhibitors in patients with recurrent and refractory OC are not satisfactory, which is far from the role of PARP inhibitors and immunotherapy in the treatment of metastatic and recurrent cervical cancer. At present, we often put PD-1/PD-L1 inhibitors in the post-treatment of OC. When other drugs are not effective, PD-1/PD-L1 inhibitors can be used alone or in combination with other drugs according to the patient’s genetic status.

## Data Availability

Publicly available datasets were analyzed in this study. This data can be found here: The datasets presented in this study can be found in online repositories. The names of the repository/repositories and accession number(s) can be found below: PubMed, Embase, and the Cochrane Library.

## Supplementary material

SUPPLEMENTARY FIGURE S1
Forest plots of the pooled mild-moderate TRAEs rate **(A)**, and severe TRAEs rate **(B)**.

SUPPLEMENTARY FIGURE S2
Begg’s publication bias funnel plots. **(A)** ORR; **(B)** DCR; **(C)** OS; **(D)** PFS; **(E)** TRARs; **(F)** iAEs.

SUPPLEMENTARY FIGURE S3
Egger’s publication bias funnel plots. **(A)** ORR; **(B)** DCR; **(C)** OS; **(D)** PFS; **(E)** TRARs; **(F)** iAEs.

SUPPLEMENTARY FIGURE S4
Sensitivity analysis for the meta-analysis. **(A)** ORR; **(B)** DCR; **(C)** OS; **(D)** PFS; **(E)** TRARs; **(F)** iAEs.

## References

[B1] Boustani J. Lecoester B. Baude J. Latour C. Adotevi O. Mirjolet C. (2021). Anti-PD-1/Anti-PD-L1 drugs and radiation therapy: Combinations and optimization strategies. Cancers (Basel) 13 (19), 4893. 10.3390/cancers13194893 34638376 PMC8508444

[B2] Brahmer J. R. Tykodi S. S. Chow L. Q. Hwu W. J. Topalian S. L. Hwu P. (2012). Safety and activity of anti-PD-L1 antibody in patients with advanced cancer. N. Engl. J. Med. 366 (26), 2455–2465. 10.1056/NEJMoa1200694 22658128 PMC3563263

[B3] Bray F. Ferlay J. Soerjomataram I. Siegel R. L. Torre L. A. Jemal A. (2020). Global cancer statistics 2018: GLOBOCAN estimates of incidence and mortality worldwide for 36 cancers in 185 countries. CA Cancer J. Clin. 70 (4), 313. 10.3322/caac.21492 30207593

[B4] Chae C. S. Teran-Cabanillas E. Cubillos-Ruiz J. R. (2017). Dendritic cell rehab: New strategies to unleash therapeutic immunity in ovarian cancer. Cancer Immunol. Immunother. 66 (8), 969–977. 10.1007/s00262-017-1958-2 28214928 PMC11028950

[B5] Curiel T. J. Coukos G. Zou L. Alvarez X. Cheng P. Mottram P. (2004). Specific recruitment of regulatory T cells in ovarian carcinoma fosters immune privilege and predicts reduced survival. Nat. Med. 10 (9), 942–949. 10.1038/nm1093 15322536

[B6] Desai J. Deva S. Lee J. S. Lin C. C. Yen C. J. Chao Y. (2020). Phase IA/IB study of single-agent tislelizumab, an investigational anti-PD-1 antibody, in solid tumors. J. Immunother. Cancer 8 (1), e000453. 10.1136/jitc-2019-000453 32540858 PMC7295442

[B7] Deshpande R. P. Sharma S. Watabe K. (2020). The confounders of cancer immunotherapy: Roles of lifestyle, metabolic disorders and sociological factors. Cancers (Basel) 12 (10), 2983. 10.3390/cancers12102983 33076303 PMC7602474

[B8] Disis M. L. Taylor M. H. Kelly K. Beck J. T. Gordon M. Moore K. M. (2019). Efficacy and safety of Avelumab for patients with recurrent or refractory ovarian cancer: Phase 1b results from the JAVELIN solid tumor trial. JAMA Oncol. 5 (3), 393–401. 10.1001/jamaoncol.2018.6258 30676622 PMC6439837

[B9] Global (2020). Latest global cancer data: Cancer burden rises to 19.3 million new cases and 10.0 million cancer deaths in 2020. [EB/OL] Available at; https://www.iarc.fr/fr/news .

[B10] Gordeeva O. (2018). Cancer-testis antigens: Unique cancer stem cell biomarkers and targets for cancer therapy. Semin. Cancer Biol. 53, 75–89. 10.1016/j.semcancer.2018.08.006 30171980

[B11] Hamanishi J. Mandai M. Ikeda T. Minami M. Kawaguchi A. Murayama T. (2015). Safety and antitumor activity of anti-PD-1 antibody, Nivolumab, in patients with platinum-resistant ovarian cancer. J. Clin. Oncol. 33 (34), 4015–4022. 10.1200/JCO.2015.62.3397 26351349

[B12] Hamanishi J. Takeshima N. Katsumata N. Ushijima K. Kimura T. Takeuchi S. (2021). Nivolumab versus gemcitabine or pegylated liposomal doxorubicin for patients with platinum-resistant ovarian cancer: Open-label, randomized trial in Japan (NINJA). J. Clin. Oncol. 39 (33), 3671–3681. 10.1200/JCO.21.00334 34473544 PMC8601279

[B13] Lim H. X. Kim T. S. Poh C. L. (2020). Understanding the differentiation, expansion, recruitment and suppressive activities of myeloid-derived suppressor cells in cancers. Int. J. Mol. Sci. 21 (10), 3599. 10.3390/ijms21103599 32443699 PMC7279333

[B14] Liu J. F. Gordon M. Veneris J. Braiteh F. Balmanoukian A. Eder J. P. (2019). Safety, clinical activity and biomarker assessments of atezolizumab from a Phase I study in advanced/recurrent ovarian and uterine cancers. Gynecol. Oncol. 154 (2), 314–322. 10.1016/j.ygyno.2019.05.021 31204078

[B15] Markham A. (2021). Dostarlimab: First approval. Drugs 81 (10), 1213–1219. 10.1007/s40265-021-01539-5 34106455

[B16] Matulonis U. A. Shapira-Frommer R. Santin A. D. Lisyanskaya A. S. Pignata S. Vergote I. (2019). Antitumor activity and safety of pembrolizumab in patients with advanced recurrent ovarian cancer: Results from the phase II KEYNOTE-100 study. Ann. Oncol. 30 (7), 1080–1087. 10.1093/annonc/mdz135 31046082

[B17] Mishra S. Liao W. Liu Y. Yang M. Ma C. Wu H. (2021). TGF-β and Eomes control the homeostasis of CD8+ regulatory T cells. J. Exp. Med. 218 (1), e20200030. 10.1084/jem.20200030 32991667 PMC7527976

[B18] Moher D. Liberati A. Tetzlaff J. Altman D. G. PRISMA Group (2009). Reprint-preferred reporting items for systematic reviews and meta-analyses: The PRISMA statement. Phys. Ther. 89 (9), 873–880. 10.1093/ptj/89.9.873 19723669

[B19] Normann M. C. Türzer M. Diep L. M. Oldenburg J. Gajdzik B. Solheim O. (2019). Early experiences with PD-1 inhibitor treatment of platinum resistant epithelial ovarian cancer. J. Gynecol. Oncol. 30 (4), e56. 10.3802/jgo.2019.30.e56 31074244 PMC6543107

[B20] O'Donnell J. S. Long G. V. Scolyer R. A. Teng M. W. Smyth M. J. (2017). Resistance to PD1/PDL1 checkpoint inhibition. Cancer Treat. Rev. 52, 71–81. 10.1016/j.ctrv.2016.11.007 27951441

[B21] Odunsi K. (2017). Immunotherapy in ovarian cancer. Ann. Oncol. 28, viii1–viii7. 10.1093/annonc/mdx444 29232467 PMC5834124

[B22] Ovarian (2022). National comprehensive cancer networks. Practice guidelines in oncology: Ovarian cancer. Available at: https://www.nccn.org/patients.Version1.

[B23] Pujade-Lauraine E. Fujiwara K. Ledermann J. A. Oza A. M. Kristeleit R. Ray-Coquard I. L. (2021). Avelumab alone or in combination with chemotherapy versus chemotherapy alone in platinum-resistant or platinum-refractory ovarian cancer (JAVELIN ovarian 200): An open-label, three-arm, randomised, phase 3 study. Lancet Oncol. 22 (7), 1034–1046. 10.1016/S1470-2045(21)00216-3 34143970

[B24] Puleo J. Polyak K. (2022). A Darwinian perspective on tumor immune evasion. Biochim. Biophys. Acta Rev. Cancer 1877 (1), 188671. 10.1016/j.bbcan.2021.188671 34933050 PMC8818030

[B25] Schuster H. Peper J. K. Bösmüller H. C. Rohle K. Backert L. Bilich T. (2017). The immunopeptidomic landscape of ovarian carcinomas. Proc. Natl. Acad. Sci. U. S. A. 114 (46), E9942–E9951. 10.1073/pnas.1707658114 29093164 PMC5699044

[B26] Shimokawa M. Kogawa T. Shimada T. Saito T. Kumagai H. Ohki M. (2018). Overall survival and post-progression survival are potent endpoint in phase III trials of second/third-line chemotherapy for advanced or recurrent epithelial ovarian cancer. J. Cancer 9 (5), 872–879. 10.7150/jca.17664 29581765 PMC5868151

[B27] Siegel R. L. Miller K. D. Fuchs H. E. Jemal A. (2021). Cancer statistics, 2021. CA Cancer J. Clin. 71 (4), 359. 10.3322/caac.21654 33433946

[B28] Topalian S. L. Taube J. M. Pardoll D. M. (2020). Neoadjuvant checkpoint blockade for cancer immunotherapy. Science 367 (6477), eaax0182. 10.1126/science.aax0182 32001626 PMC7789854

[B29] Varga A. Piha-Paul S. Ott P. A. Mehnert J. M. Berton-Rigaud D. Morosky A. (2019). Pembrolizumab in patients with programmed death ligand 1-positive advanced ovarian cancer: Analysis of KEYNOTE-028. Gynecol. Oncol. 152 (2), 243–250. 10.1016/j.ygyno.2018.11.017 30522700

[B30] Zhai L. Bell A. Ladomersky E. Lauing K. L. Bollu L. Sosman J. A. (2020). Immunosuppressive Ido in cancer: Mechanisms of action, animal models, and targeting strategies. Front. Immunol. 11, 1185. 10.3389/fimmu.2020.01185 32612606 PMC7308527

[B31] Zhu J. Yan L. Wang Q. (2021). Efficacy of PD-1/PD-L1 inhibitors in ovarian cancer: A single-arm meta-analysis. J. Ovarian Res. 14 (1), 112. 10.1186/s13048-021-00862-5 34454562 PMC8403414

