# Supplementary figures and images for: Efficacy and safety of PD-1/PD-L1 inhibitors in the treatment of recurrent and refractory ovarian cancer: A systematic review and a meta-analysis
Source: Front Pharmacol. 2023 Mar 13;14:1111061. doi: 10.3389/fphar.2023.1111061 (PMC10042289; doi:10.3389/fphar.2023.1111061)

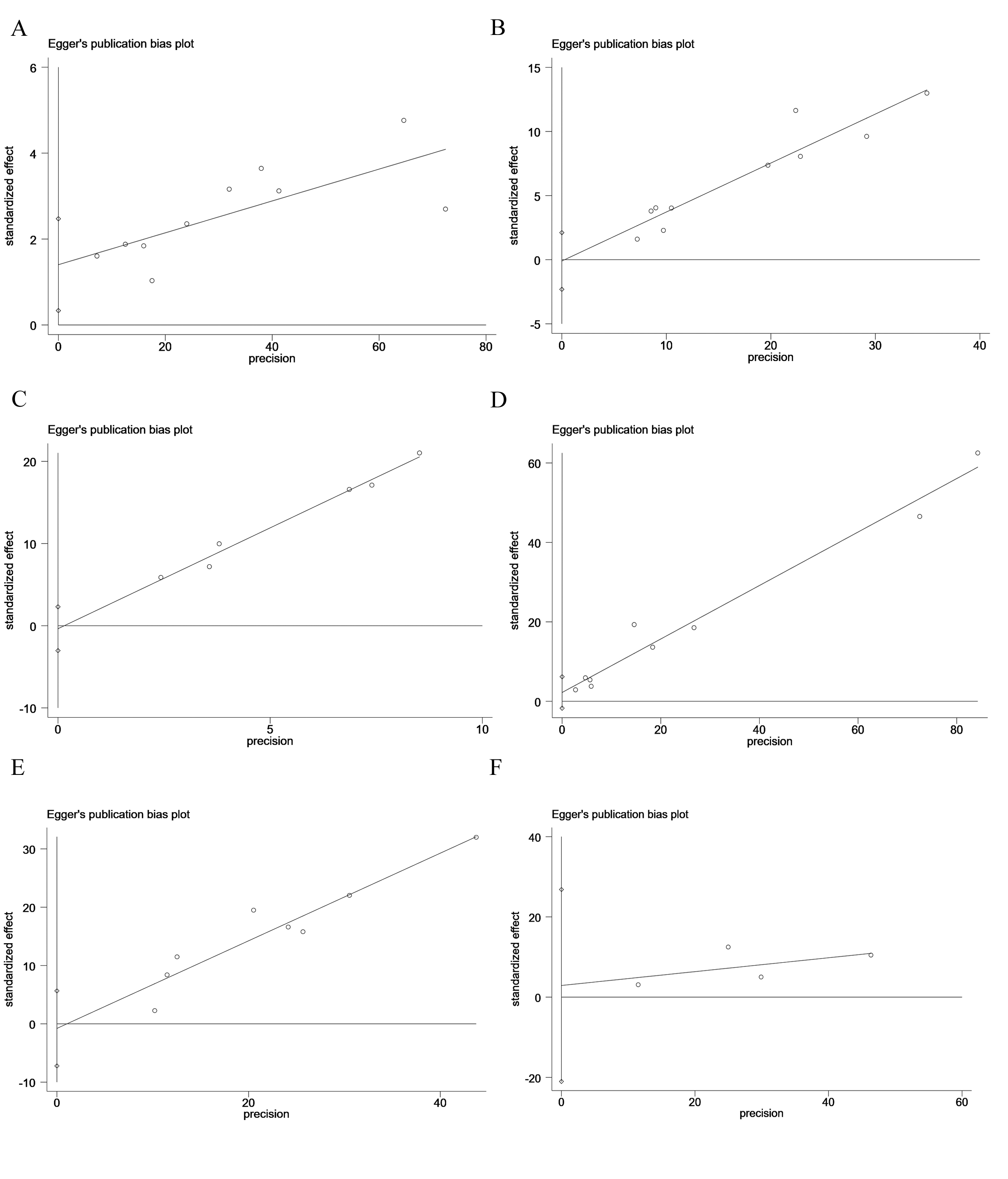

Supplement: Supplementary file 1 [file Image3.JPEG]

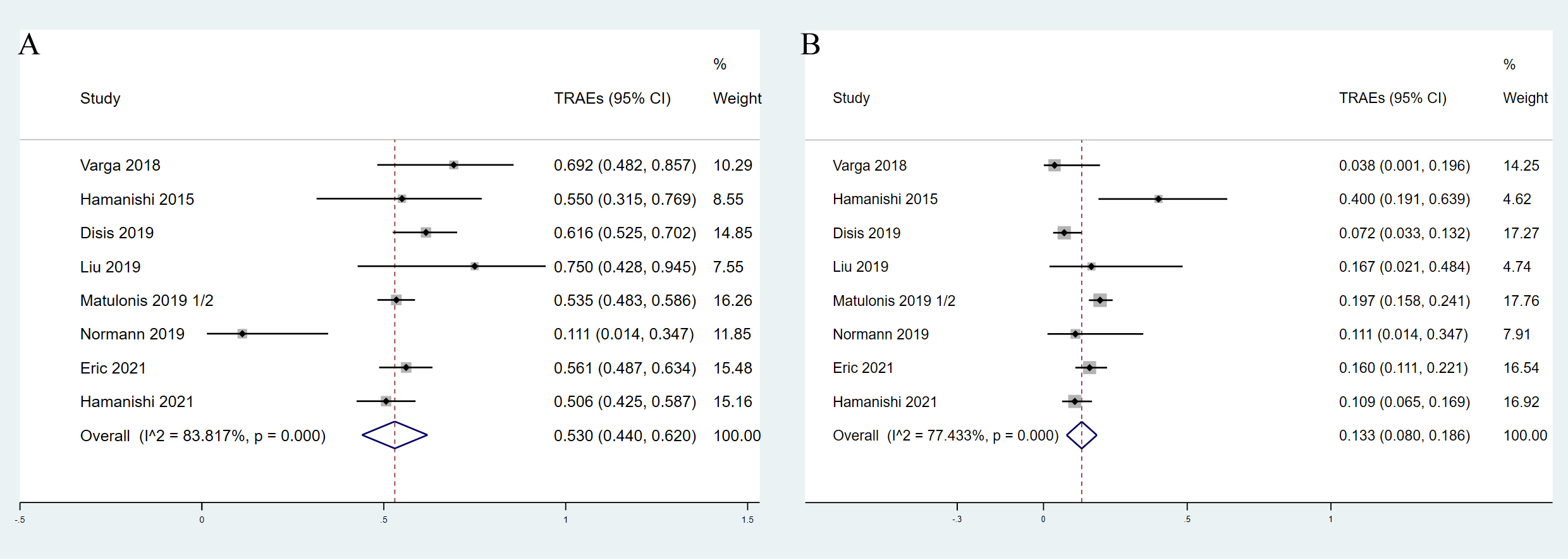

Supplement: Supplementary file 2 [file Image1.JPEG]

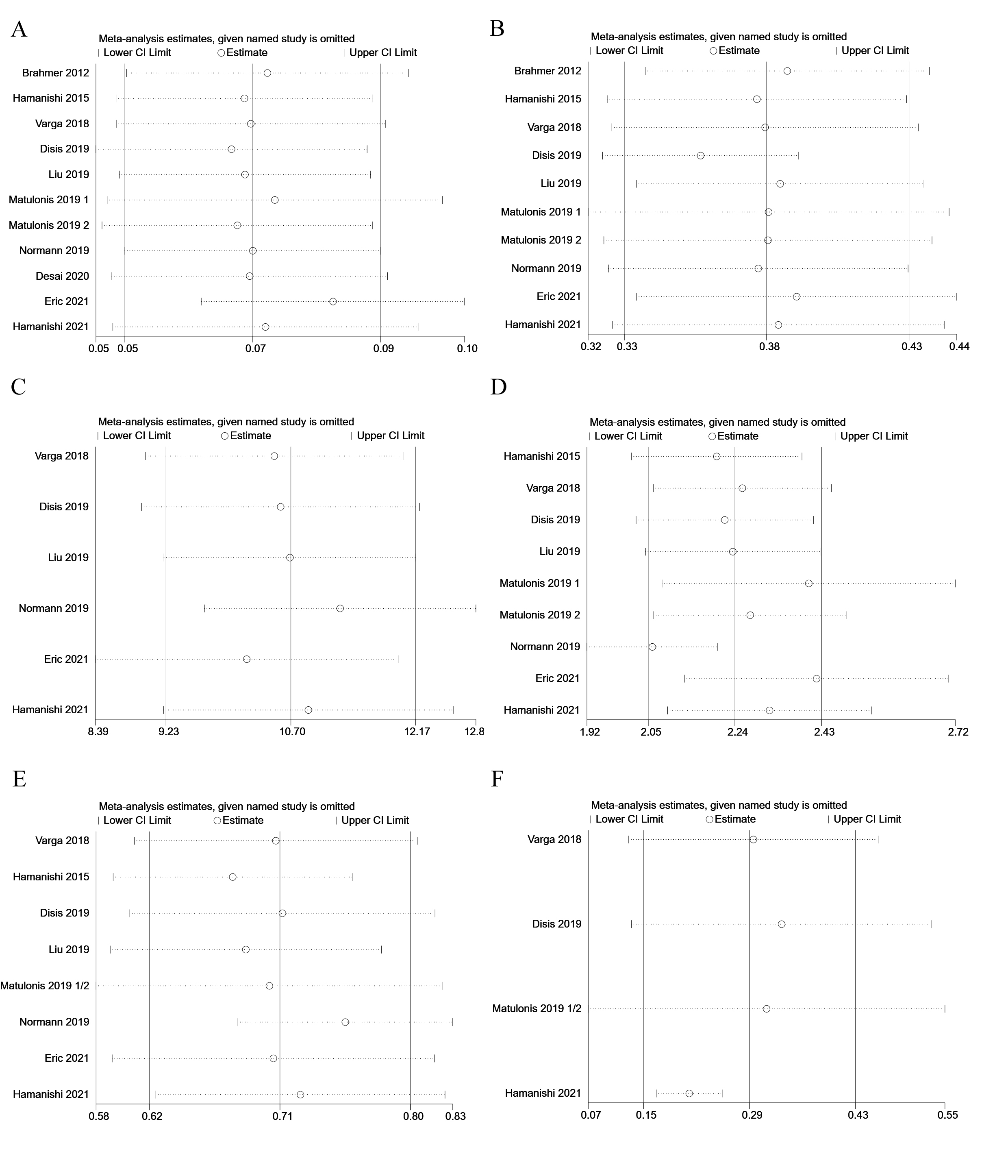

Supplement: Supplementary file 3 [file Image4.JPEG]

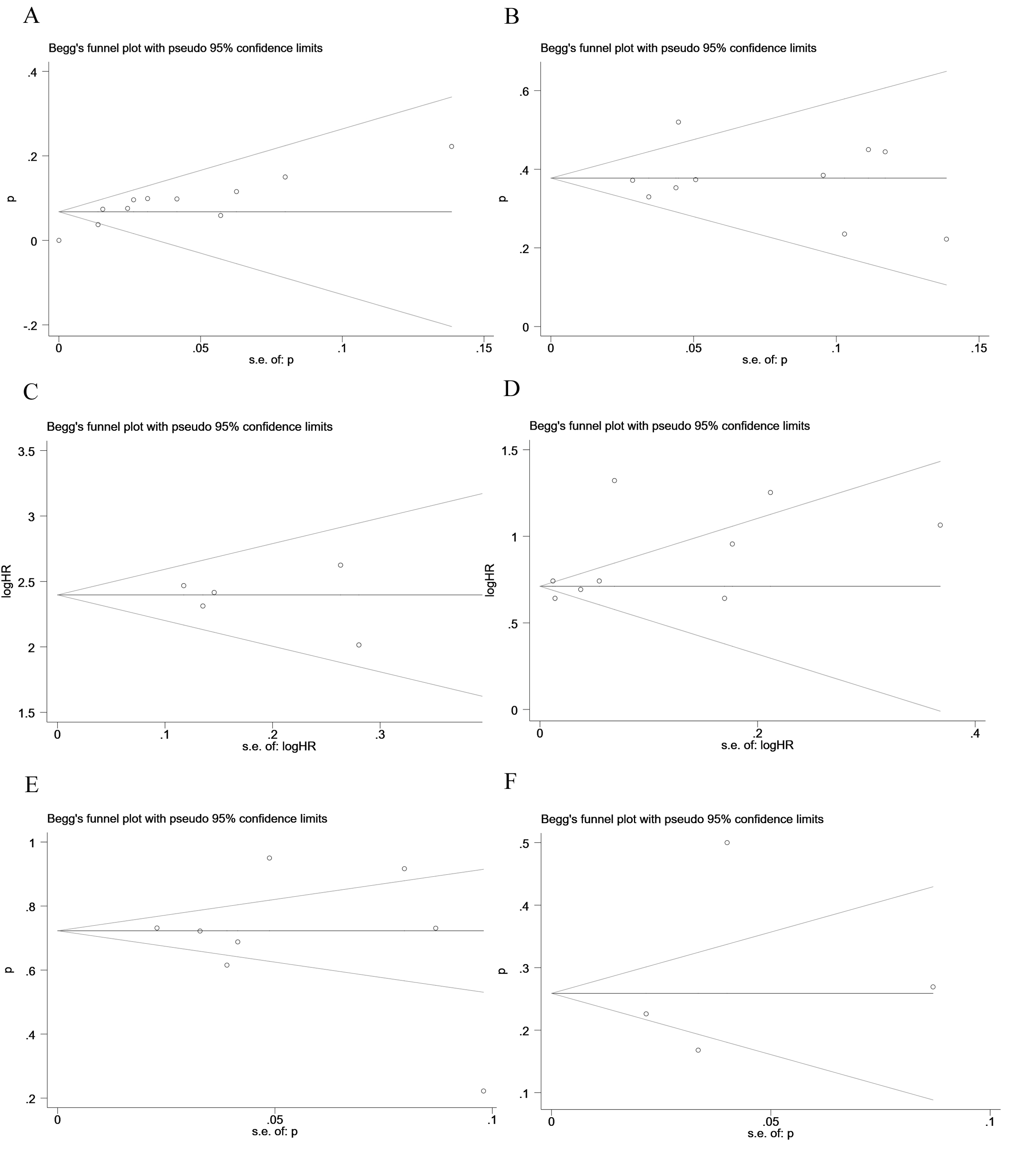

Supplement: Supplementary file 4 [file Image2.JPEG]
